# Maternal exposure to the thyroperoxidase-inhibiting pesticide amitrole induces hypothyroidism and developmental neurotoxicity in the rat brain

**DOI:** 10.3389/fendo.2026.1823237

**Published:** 2026-05-12

**Authors:** Louise Ramhøj, Jacob Ardenkjær-Skinnerup, Nichlas Davidsen, Jermaine Ford, Carter Kuehn, Caroline Frädrich, Josef Köhrle, Mary Gilbert, Terje Svingen, Marta Axelstad

**Affiliations:** 1 National Food Institute, Technical University of Denmark, Kgs. Lyngby, Denmark; 2 Center for Computational Toxicology and Exposure, Office of Research and Development, U.S. Environmental Protection Agency, Research Triangle Park, NC, United States; 3 Charité - Universitätsmedizin Berlin, corporate member of Freie Universität Berlin, Humboldt-Universität zu Berlin, Institut für Experimentelle Endokrinologie, Berlin, Germany; 4 Center for Public Health and Environmental Assessment, Office of Research and Development, U.S. Environmental Protection Agency, Research Triangle Park, NC, United States

**Keywords:** DNT, endocrine disruption, heterotopia, hypothyroidism, thyroid disruption, thyroid peroxidase

## Abstract

Thyroid hormone (TH) system-disrupting chemicals pose significant risks to human health and the environment, highlighting the urgent need for improved toxicological testing methods. A major concern is that environmental chemicals may induce developmental neurotoxicity by compromising TH signaling during critical life stages. In this study, we evaluated the thyroidal and neurotoxic effects of two compounds that interfere with TH signaling by inhibiting TH synthesis in the thyroid gland. Methimazole (MMI) is a pharmaceutical specifically designed to treat hyperthyroidism by inhibiting thyroperoxidase (TPO), while the herbicide amitrole has unintentional TPO-inhibiting properties. Pregnant and lactating rat dams were exposed to control (corn oil), 8 or 16 mg/kg body weight/day MMI, or 25 or 50 mg/kg body weight/day amitrole from gestation day 7 to postnatal day 16. MMI and amitrole induced fetal and postnatal hypothyroidism in offspring. Both compounds induced similar reductions in serum and brain TH concentrations and induced a brain malformation, periventricular heterotopia, tied to compromised TH signaling. Our results reinforce concerns over the potential for exposure to environmental chemicals with TH system-disrupting properties to negatively impact the developing brain in vertebrates.

## 1 Introduction

Thyroid hormone (TH) system-disrupting chemicals can interfere with TH signaling, leading to endocrine dysfunction and downstream effects on brain development. Severe TH deficiency profoundly affects brain structure and function in both humans and animals. Even mild deficiencies are concerning; in pregnant women, serum free thyroxine (T4) concentrations at the lower end of the reference interval have been linked to suboptimal neurodevelopment in their children (1–3). Reported outcomes include reduced Intelligence Quotient (IQ), altered brain morphology, impaired motor development, and increased risk of neurobehavioral disorders such as autism spectrum disorders and schizophrenia (1–3). In rats, the effects of mild TH insufficiency are less clear, and evaluating developmental neurotoxicity from environmental chemicals that moderately reduce serum and brain TH concentrations remains challenging (4–12).

Chemicals can disrupt the TH system by inhibiting thyroperoxidase (TPO), a key enzyme required for TH biosynthesis in the thyroid gland. TPO serves to catalyze iodide oxidation and iodinate tyrosine residues on thyroglobulin, and to couple iodotyrosine residues to form the THs thyroxine (T4) and 3,3′,5-tri-iodothyronine (T3) (13). *In vitro* studies have shown that environmental chemicals can inhibit TPO (14, 15). The pharmaceuticals propylthiouracil (PTU) and methimazole (MMI) are used to treat hyperthyroidism by inhibition of TPO and are also widely used as model compounds in animal studies investigating the critical role of TH signaling in neurodevelopment. *In vivo* TPO inhibition can impair TH synthesis in the thyroid gland. This leads to a reduction in circulating levels of TH and supply of TH to target tissues, including the brain. Such action during development can disrupt critical TH-dependent processes and lead to long-lasting neurological impairments (16–18).

One clear adverse consequence of TPO inhibition is the induction of periventricular heterotopia, a brain malformation composed of clusters of misplaced neurons appearing in the corpus callosum of rat offspring (19–22). Heterotopia represents a defect in neuronal migration, forming early and persisting throughout the life of the animal. Heterotopia forms if sustained and sufficient brain T3 deficiency occurs from approximately gestation day (GD) 19 to the early postnatal period (19, 21–23). Although most work characterizing this brain defect has focused on PTU (7, 10, 19–22, 24–26), MMI delivered to pregnant rat dams from early gestation has also been shown to induce heterotopia (19, 25, 27). However, detailed developmental dose–response data linking serum and brain TH levels to heterotopia are lacking for MMI and TPO-inhibiting environmental chemicals.

To begin to address the possibility that TPO inhibition by environmental contaminants may also lead to deleterious effects in brain, the triazole herbicide amitrole was investigated. Amitrole was selected due to its demonstrated *in vitro* TPO-inhibitory activity and ability to reduce serum TH *in vivo* (28, 29). In a preliminary report, we recently demonstrated that maternal exposure to amitrole induced hypothyroidism in dams and offspring, and heterotopia in pups (30). This represented the first report of an environmental contaminant with TPO-inhibiting properties reducing serum TH and impairing brain development. Here, we replicate and expand upon these findings with amitrole and MMI by examining dose–response relationships at gestational and early postnatal time points to more fully characterize the serum and brain TH levels and heterotopia formation.

## 2 Materials and methods

Work presented herein is a continuation of a reproductive toxicity study previously described in full (29). Previous reports include reproductive, thyroid-related, and neurotoxic endpoints (29, 31) and serum chemical and steroid hormone concentrations, along with testes histology and transcriptomes (32, 33). New data reported herein include complete TH-related and toxicity data from GD21 fetuses and their pregnant mothers, as well as offspring TH in serum and brain in the fetus, newborn, and neonate along with assessments of heterotopia.

### 2.1 Animals

**Figure 1:**
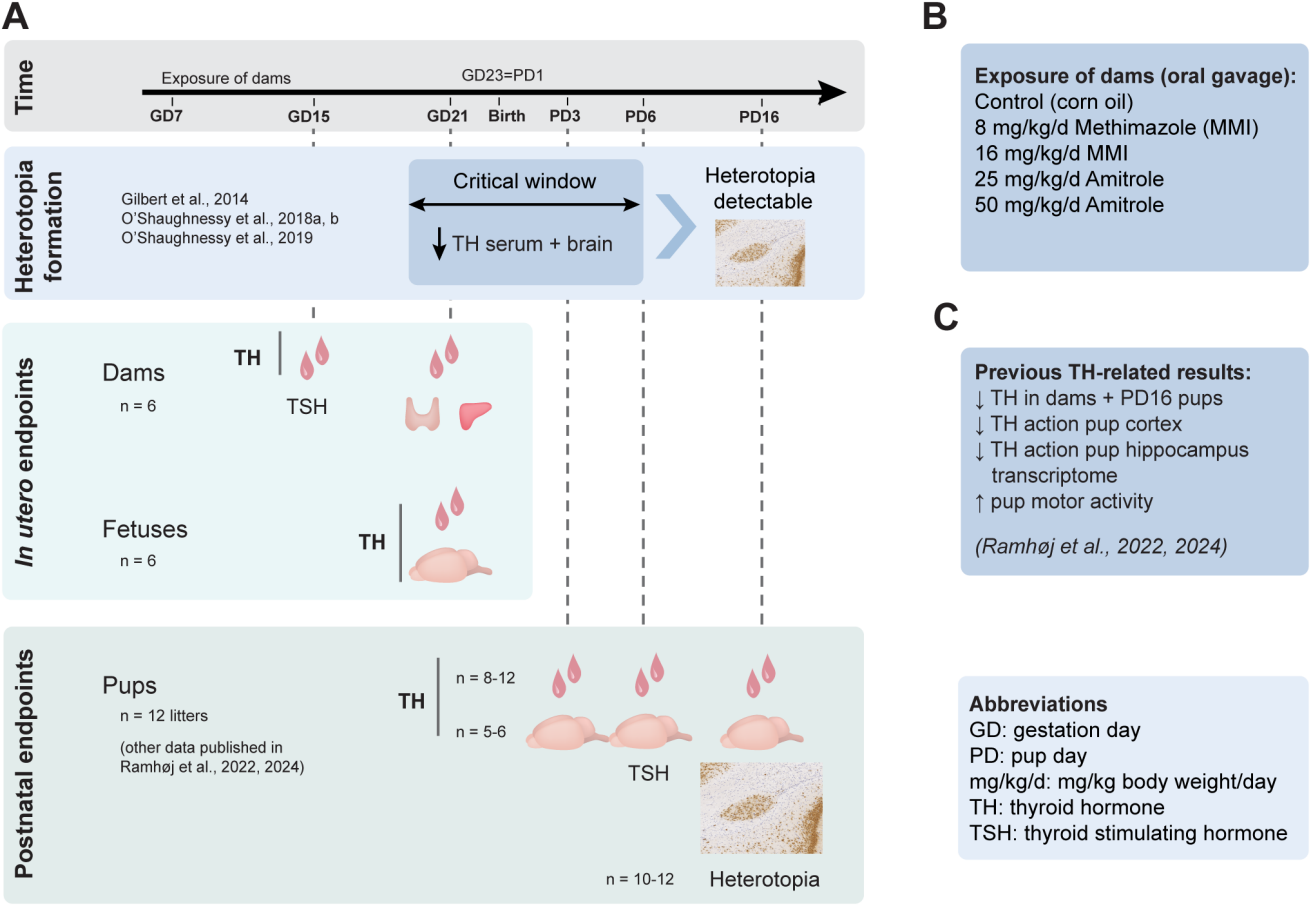
Study design and previous results. Pregnant rat dams were exposed to two doses of MMI (8 or 16 mg/kg/d) or amitrole (25 or 50 mg/kg/d) from gestation day (GD) 7 to pup day (PD) 16 **(A, B)**. Periventricular heterotopia is a malformation of the brain seen in the corpus callosum of rat offspring following developmental TH deficiency during a critical window for TH deficiency approximately between GD19 and PD6 (20–22, 42). THs were measured in tongue blood from dams on GD15 (RIA, T4 LOQ = 4 nM ~ 3.1 ng/mL) and in serum (trunk blood) from dams and fetuses as well as brain of fetuses on GD21 (LC-MS/MS, serum LOQ = 0.02 ng/mL) (*n* = 6) **(A)**. A separate group of dams (*n* = 11–12) gave birth on GD23 = PD1, with THs measured in offspring serum and brain tissue at PD3, PD6, and PD16. Any formation of periventricular heterotopia was assessed in brains of PD16 male rat offspring (*n* = 10–12). Data from this study have previously been published in (29, 31–33) **(C)**. These studies showed effects in the postnatal offspring, the TH system, testes development, and TH action in the cortex and hippocampus and adverse effects on motor activity. Doses are given in mg/kg body weight/day (d). GD, gestation day; LC-MS/MS, liquid chromatography/tandem mass spectrometry; MMI, methimazole; PD, pup day; RIA, radioimmunoassay; T3, tri-iodothyronine; T4, thyroxine; TH, thyroid hormones; TSH, thyroid-stimulating hormone.

**Figure 1** gives a graphical overview of the rat exposure study and endpoints measured in this study, with detailed description available in (29). Briefly, time-mated Sprague–Dawley rat dams [240 ± 30 g, Crl: CD(SD) bred by Charles River Europe, distributed by Scanbur, Denmark] were received on GD3. The day of plug detection was defined as GD1 and expected day of delivery (GD23) was designated as pup day (PD) 1. This timing leads GD21 dissections to take place approximately 36 h before expected birth and PD3 pups most likely fall between 48 and 58 h old. Dams were exposed to MMI (CAS no: 60-56-0, Sigma-Aldrich 301507-5G, batch: WXBC9951 V, purity 99.5%) or amitrole (3-amino-1H-1,2,4-triazole, CAS no: 61-82-5, Sigma-Aldrich 8144950100, batch: S7075495 925, purity 99.7%) by oral gavage from GD7 to PD16, except on the day of parturition; i.e., offspring were exposed via placenta until birth, then via breast milk until PD16. The animals were pseudo-randomly distributed into five exposure groups with similar body weight distribution: control (vehicle, corn oil, Sigma-Aldrich, cat. no. C82677 – 2.5 L), 8 mg/kg body weight (bw)/day (d) MMI, 16 mg/kg/d MMI, 25 mg/kg/d amitrole, and 50 mg/kg/d amitrole. Six dams per group were included in the gestational cohort while the postnatal cohort consisted of 11–12 dams/litters per group. The body weight of dams was recorded every morning and dosing was given at a volume of 2 mL/kg bw/d. The animals had *ad libitum* access to acidified tap water and a standard Altromin 1314 soy- and alfalfa-free diet (Altromin GmbH, Lafe, Germany) containing 1.52 mg/kg iodine and 0.26 mg/kg selenium. Dams with their litters were housed in semi-transparent polysulfone cages (PSU 80-1291HOOSU Type III, Tecniplast S.p.A, Buguggiate, Italy) with aspen wood chip bedding (Tapvei, Gentofte, Denmark) and Enviro dry nesting material and aspen wood shelters from Brogaarden, Lynge, Denmark). Environmental conditions were controlled by ScanClime coupled to ScanTainers (both from Scanbur, Karlslunde, Denmark): 55% ± 5% humidity, 21 ± 1 °C, air change 50 times per hour, and a reversed light/dark cycle.

This study was overseen by the in-house Animal Welfare Committee for animal care and use and ethical approval was given by the Danish Animal Experiments Inspectorate, authorization number 2020-15-0201-00539.

### 2.2 Necropsy, organ weights, and brain samples

Six dams per group were weighed and euthanized by decapitation under CO_2_/O_2_ anesthesia on GD21. Dam livers and thyroid glands were excised and weighed. Uteri were excised, fetuses were extracted, and the number of resorptions and implantations was counted. Fetuses were sexed, weighed, and decapitated, and samples were retrieved as described below [data on fetal serum MMI and amitrole concentrations and steroid hormones have been in published (32)].

Eleven to 12 dams per group gave birth to viable litters. From each litter, when available, one to two male and one to two female pups were decapitated on PD3 and PD6 and two males were euthanized by decapitation under CO_2_/O_2_ anesthesia on PD16. Forebrain samples were collected for TH quantification from one male fetus/pup per litter on GD21, PD3, PD6, and PD16. On PD3, the sample consisted of mostly hippocampus and cortex; on PD6 and PD16, the sample consisted of mostly anterior cortex. From one PD16 male pup per litter, the brain was extracted and immersion-fixed in neutral buffered 10% formalin for 5 days at room temperature. During the first 24 h, containers were placed on a tube roller (CAPP CRR-08X, AHN Biotechnologie GmbH).

### 2.3 Serum samples

Dam serum samples were taken as tongue blood on GD15 and trunk blood on GD21. Fetal trunk blood was collected and pooled by sex within each litter on GD21 and from one to two male and one to two female pups per litter on PD3 and PD6, and from two males per litter on PD16. Samples were collected in Eppendorf tubes without heparin, placed on ice <1 h and centrifuged at 4,000 rpm for 10 minutes (~2,400 x g) at 4°C (Eppendorf 5403 centrifuge, Eppendorf) and stored at −80 °C until analysis.

### 2.4 Thyroid-stimulating hormone

Serum thyroid-stimulating hormone (TSH) was measured in GD15 dams and PD6 male and female offspring serum (one to two pups pooled per sex) using a Milliplex MAP rat pituitary magnetic bead panel (RPTMAG-86 K, EMD Millipore, Darmstadt, Germany) following the manufacturer’s instructions. The intra-assay coefficient of variation was <10.5%, and the limit of quantification was 3.2 pg/mL as described in (29).

### 2.5 Serum TH in GD15 dams by radioimmunoassay

Serum total T3 and total T4 was measured in GD15 dam serum by validated radioimmunoassay (RIA) and with modifications to reduce the lower limit of quantification (LOQ) as described in (29). Briefly, kits for T4 (RIA4524, DRG Instruments, Marburg Germany) and T3 (RIA4525, DRG Instruments, Marburg, Germany) were used with double standard and sample volumes, and the standard curve was extended by diluting standard calibrators. The LOQs were 4.0 nM T4 and 0.2 nM T3. The intra-assay coefficients of variation were <9%.

### 2.6 Sample preparation for serum TH by liquid chromatography/tandem mass spectrometry (LC-MS/MS)

Total T3 and T4 concentrations were measured in serum from GD21 dams and fetuses as well as PD3, PD6, and PD16 offspring (for PD16 only males) by liquid chromatography/tandem mass spectrometry (LC-MS/MS), providing a lower LOQ to enhance the detection of low hormone concentrations in fetuses and high-dose groups [see (34, 35)]. Serum from dam (20 μL) and fetal/pup (40 μL) samples was aliquoted into Eppendorf tubes and spiked with stable isotope internal standard containing T3-^13^C_6_, T4-^13^C_6_, rT3-^13^C_6_, and 3,3′-T2-^13^C_6_ dissolved in methanol. Samples received equal volumes of 1 N HCl, five times the volume of water, and, finally, three times the volume of 50:50 water/acetonitrile (v/v) with 0.1% formic acid. The samples were briefly mixed, and the proteins were hydrolyzed at 37 °C for 2 h, cooled to room temperature, and diluted with 0.1% aqueous acetic acid to a total volume of 400 μL dam and 800 μL fetal. Samples were vortexed and solid phase extracted (SPE) using an Evolute CX (30 mg) SPE plate (Biotage, Charlotte, North Carolina, USA) with a vacuum manifold. Cartridges were conditioned with 1.0 mL of methanol followed by 1.0 mL of aqueous 0.1% formic acid; samples were applied, followed by a 1.0-mL aqueous 0.1% formic acid wash, and a second wash with 1.0 mL of methanol. THs were eluted using methanol with 4% ammonium hydroxide into a collection plate and evaporated to dryness using nitrogen. The dried samples were reconstituted with 100 μL of 25:75 water/methanol (v/v) with 0.1% formic acid for quantification of TH as described below.

### 2.7 Sample preparation for brain TH by LC-MS/MS

Brain TH levels were measured in male brains from GD21, PD3, PD6, and PD16 according to procedures outlined in (34) with some modifications as described below. Hormones were extracted from brain samples with 8 µL of methanol per milligram of tissue using an Omini BeadRuptor homogenizer. Samples were briefly vortexed and a 200-µL aliquot was transferred into a 2-mL Eppendorf tube. Internal standards, which included 13C isotope-labeled versions of MIT, DIT, 3,3′-T2, T3, rT3, and T4 at a concentration of 100 ng/mL, were added along with 50 μL of Cleanascite (Biotech Support Group, New Jersey, USA), a non-ionic solid-phase lipid removal agent. The samples were then incubated at room temperature for 10 min before centrifugation at 16,000 rpm for 5 min. The supernatant was carefully removed and placed into a new 2-mL Eppendorf tube. An additional 200 µL of methanol was added to the residual pellet, which was re-homogenized and centrifuged again, allowing the supernatants to be combined. To ensure the release of THs from any remaining proteins and sufficiently acidify the solution for optimal interaction with the solid-phase extraction (SPE) sorbent, 500 µL of 0.05 N hydrochloric acid was added to the combined supernatants. The THs were then extracted using a Biotage Evolute CX cartridge (30 mg/1 mL) (Charlotte, North Carolina, USA). The cartridges were conditioned with 1 mL of methanol and equilibrated with 1 mL of water, and samples were loaded at a flow rate of 1 mL/min. Interferences were washed from the cartridge using 1 mL of 0.1% acetic acid in water, 1 mL of methanol, 1 mL of 1% formic acid in ethyl acetate, and 500 µL of methanol. The cartridges were allowed to dry under vacuum for 3 min and THs were eluted in 150 µL of 4% ammonium hydroxide in methanol (×3). Eluents were completely dried using a gentle stream of nitrogen, and dried extracts were then reconstituted in 100 µL of a methanol:water solution (1:1, v/v) with 2% formic acid for quantification of TH as described below.

### 2.8 Quantification of TH in serum and brain by LC-MS/MS

Serum and brain TH were quantified by LC-MS/MS using an AB Sciex Exion AC UHPLC coupled to a QTRAP 6500+ Linear Ion Trap mass spectrometer system (Framingham, Massachusetts, USA). THs were separated on a Raptor Biphenyl HPLC column (Restek Corporation, Bellefonte, Pennsylvania, USA). Mobile phase A consisted of water and mobile phase B consisted of methanol both with 0.1% formic acid. Quantitation was performed based on isotope dilution from a solvent-based calibration curve ranging from 0.01 to 100 ng/mL. Calibration curves comprised at least five sequential points, and the correlation coefficients of the curves were ≥0.998. Qualitative confirmation was evaluated based on relative ion ratio calculations and relative retention times. Ion ratios were calculated by monitoring two ion transitions per analyte and comparing the relative ion ratio of the samples to the standards. The retention time of each analyte peak was qualitatively identified based on retention time relative to the internal standard and calibration standard. The LOQ for each analyte was set to the concentration of the lowest calibration standard that gave an acceptable ion ratio and an acceptable recovery of ±30% of the spiked amount, 0.01 ng/mL for both T3 and T4. Each sample batch contained a method blank, a laboratory control sample (spiked phosphate buffered saline), and a continuing calibration verification sample prepared in the solvent. The LOQ for both serum T4 and T3 was 0.02 ng/mL, and the lower limit of detection (LOD) was 0.01 ng/mL. For brain, the LOQ was 0.02 ng/g and the LOD was 0.01 ng/g for both T3 and T4. Samples below the LOD were assigned a value equal to the LOD.

### 2.9 Brain sectioning and immunohistochemistry

The formalin-fixed PD16 brains were coronally blocked, and a thick section containing the hippocampus was processed in an Excelsior AS Tissue Processor (Thermo Scientific, United Kingdom) and embedded in paraffin. For assessment of volumes of periventricular heterotopia, the brains were sectioned coronally at 10 µm. Every third section was collected from anterior to posterior hippocampus [collected sections spanned from approximately plate 26 to 40 of (36)], yielding ~60 sections per brain for heterotopia assessment. For immunohistochemical (IHC) staining of NeuN, brain sections were deparaffinized and targets were retrieved in a PT link (PT200, Dako, Denmark) with high-pH EnVision FLEX Target retrieval solution (K8004, Dako) diluted to 0.25% in deionized water. The sections were warmed from 65 °C to 97 °C for 18 min, incubated at 97 °C for 20 min, and cooled to 65 °C for 35 min. Sections were rinsed in rinse buffer [0.9% NaCl, 0.2% Triton X-100 (Millipore), and 5% PBS buffer] for approximately 5 min before being transferred to an intelliPATH FLX automated IHC stainer (IPS0001INTL, Biocare Medical, USA). Using the stainer, the sections were blocked with 1% bovine serum albumin (BSA, P6155, Biowest) in PBS buffer for 30 min then incubated with 1:1,500 NeuN primary antibody (MAP377 Neuronal Nuclei, Sigma-Aldrich) in 1% BSA in PBS for 30 min at 21 °C. Endogenous peroxidase was blocked with 3% H_2_O_2_ for 10 min and sections were incubated with Envision+ System-HRP Labeled Polymer Anti-Mouse (K4001, Dako) for 30 min, then stained with the Liquid DAB+ Substrate Chromogen System (K3468, Dako) solution for 10 min. Sections were counterstained with Mayer’s hematoxylin (AMPQ00254.0500, Ampliqon) and coverslipped with Eukitt Mounting Medium (03989, Sigma-Aldrich).

### 2.10 Imaging and periventricular heterotopia assessment

Stained slides were imaged at 40× on a NanoZoomer 2.0 HT slide scanner (NanoZoomer Digital Pathology, Hamamatsu). The presence of heterotopia was assessed in the corpus callosum (from the midline and to inferior to frontal cortex area 2). A heterotopia was defined as a cluster of a minimum of five large NeuN-positive cells present on at least two adjacent sections (20 µm between two adjacent sections). If a section adjacent to a heterotopia-containing section contained less than three large NeuN-positive cells, these were not considered part of the heterotopia. Areas of all heterotopia were determined at 10× magnification with the NDP.view 2.9.29 software (Hamamatsu Photonics K.K., Japan). Heterotopia volumes were calculated by multiplying the sum of all areas for each animal with 30 µm (the thickness of one section plus the distance to the next section). In a few instances, one or more sections were missing for technical reasons; these were dealt with as follows. When one or more sections were missing and there were heterotopia on the adjacent sections, the heterotopia area of the missing section was estimated to be the average area of the adjacent sections. When there was only a heterotopia on one of the adjacent sections and its area was ≥5,000 µm^2^, the heterotopia of the missing section was estimated as half of the area of the adjacent heterotopia. If the adjacent heterotopia was <5,000 µm^2^, it was estimated that a heterotopia was not present on the missing section. In addition to calculating a total heterotopia volume per animal, heterotopia formation was also assessed as the rostral–caudal length of heterotopia. The total number of sections with heterotopia (one section counted twice when there were heterotopia in both hemispheres) was multiplied by 30 µm and divided by two.

### 2.11 Statistical analysis

All continuous endpoints were analyzed by General Linear Models (GLM) to test for exposure-related effects compared to the control group. This was done both with all exposure groups in the same analysis and for the control and MMI or amitrole-exposed groups separately. These two approaches yielded largely similar results. Data were log-transformed when requirements for normal distribution and homogeneity of variance were not met. Dunnett’s *post-hoc* test was used to account for multiple comparisons with the statistical significance level set at 0.05. The nonparametric Kruskal–Wallis test with Dunn’s *post-hoc* test was used when parametric assumptions were not met. Covariates were included in the statistical analysis of fetal weight (number of pups) and for liver and thyroid weights (body weight). Litter effects were accounted for throughout by including the litter as an independent random nested factor in the analysis of fetal weights, by pooling serum within litter and by only analyzing one pup per litter. Volume and length of periventricular heterotopia were analyzed for effect differences by a Poisson regression model with adjustment for multiple testing according to Holm–Sidak (37). Post-implantation loss (prenatal death) was analyzed by the non-parametric Kruskal–Wallis test. SAS Enterprise v8.3 (SAS institute, NC, US) and GraphPad Prism 9 (GraphPad Software, CA, US) were used for all statistical analyses.

## 3 Results

### 3.1 Toxicity data from the prenatal cohort

In the cohort of animals used for the prenatal study, there were no effects on post-implantation loss, litter size, or sex ratio. The dams exposed to the high dose of MMI gained less weight during gestation. On study termination (GD21), a significant decrease in body and liver weight (both absolute and relative to body weight) was seen (**Supplementary Table S1**). Fetal body weights appeared reduced, most noticeably in the high-dose groups, with males exposed to high-dose amitrole displaying a statistically significant body weight reduction relative to controls.

Data on growth and development in dams and offspring from the cohorts giving birth were previously reported in (29). In summary, there were no signs of overt toxicity, but offspring growth was inhibited in both high-dose groups, with sporadic effects also in the low-dose MMI group (29).

### 3.2 TH system disruption in pregnant dams

In pregnant dams, dose-dependent reductions in serum T4 and T3, and elevations in TSH and thyroid gland weights were apparent for both substances (**Supplementary Table S2**). The effects were more pronounced in dams on GD21 compared to GD15 and in the high-dose groups.

### 3.3 Disruption of TH concentrations in serum of fetuses and postnatal offspring

**Figure 2:**
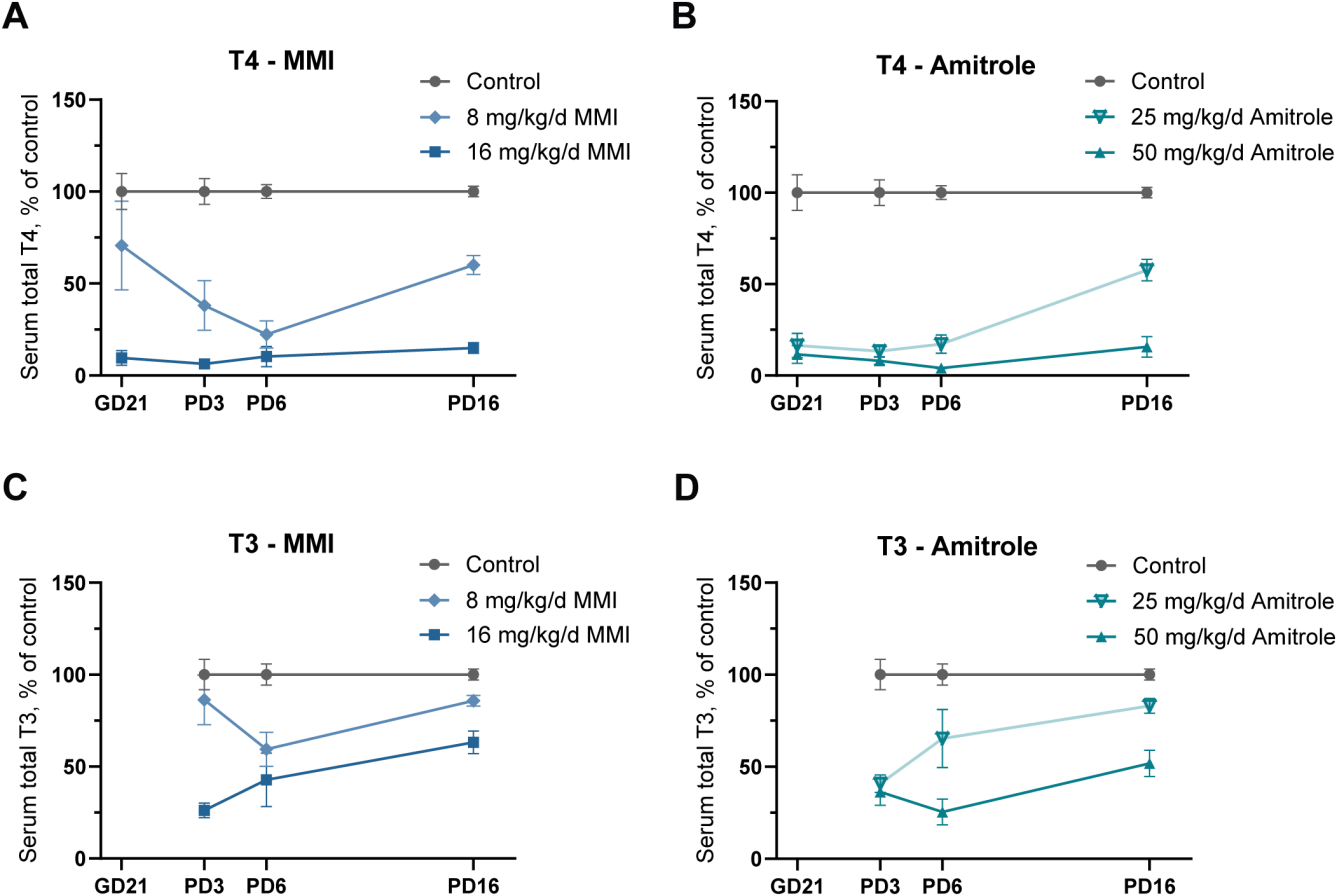
Gestational and lactational exposure to the thyroperoxidase inhibitors MMI and amitrole reduced offspring serum total T3 and T4 during development. Rat dams were exposed to two doses each of MMI (8 or 16 mg/kg/d) or amitrole (25 or 50 mg/kg/d) from GD7 to PD16, and THs were measured in serum from GD21 fetuses and PD3, PD6, and PD16 male offspring. Serum total T4 concentration expressed as a percentage of control was reduced in both MMI **(A)** and amitrole **(B)** exposed offspring. In the high-dose groups, the reductions were sustained below 20% of controls throughout development. In contrast to low-dose amitrole, low-dose MMI caused only slight and nominal reductions in serum T4 concentrations in the GD21 fetuses, while both low-dose groups had reduced serum T4 concentrations in the PD16 male offspring. Serum total T3 concentration as a percentage of control was reduced in both MMI **(C)** and amitrole **(D)** exposed offspring. Serum total T3 concentration was not quantifiable in GD21 fetuses. For PD16 serum, TH concentrations were previously measured by radioactive immunoassay (RIA) in a different serum aliquot; these data are published in (29). Data are shown as group means ± SEM. Sample sizes and indications of statistical significance are given in **Supplementary Table S2**. GD, gestation day; LC-MS/MS, liquid chromatography/tandem mass spectrometry; mg/kg/d, mg/kg body weight/day; MMI, methimazole; PD, pup day; T3, tri-iodothyronine; T4, thyroxine.

MMI and amitrole had significant effects on fetal and postnatal offspring serum TH concentrations. Male offspring serum TH profiles over developmental time are shown in **Figure 2**, as a percentage of control. Absolute TH concentrations in male and female offspring and the results of statistical analyses can be found in **Supplementary Table S2**. High-dose MMI and both doses of amitrole reduced serum T4 concentrations of offspring to below 25% of control during the fetal and early postnatal period (GD21–PD6), with some recovery evident by PD16 in the animals exposed to the low dose of amitrole (**Figures 2A, B**). In contrast, fetal serum T4 concentrations were not significantly altered in the low-dose MMI group, although high variability was seen across litters. The variability in fetal serum T4 concentration was paralleled in MMI-exposed dams, where two litters were strongly affected while the remaining four were barely impacted (**Figure 2A**). Significant reductions in serum T4 concentrations did emerge on PD3, increased by PD6, with some recovery present by PD16, matching that seen in the low-dose amitrole group at that age. Fetal serum T3 concentration was below LOQ on GD21 but was measurable in the postnatal pups. Significant reductions were observed in serum T3 concentration at all time points in the high-dose group of both MMI and amitrole. In the low-dose MMI group, serum T3 concentration did not differ from control levels on PD3, was suppressed at PD6, and recovered by PD16. Low-dose amitrole reduced serum T3 concentration at PD3 with recovery evident at PD6, and as with MMI, no difference from control was detected on PD16.

### 3.4 Disruption of TH concentration in brain in fetuses and postnatal offspring

**Figure 3:**
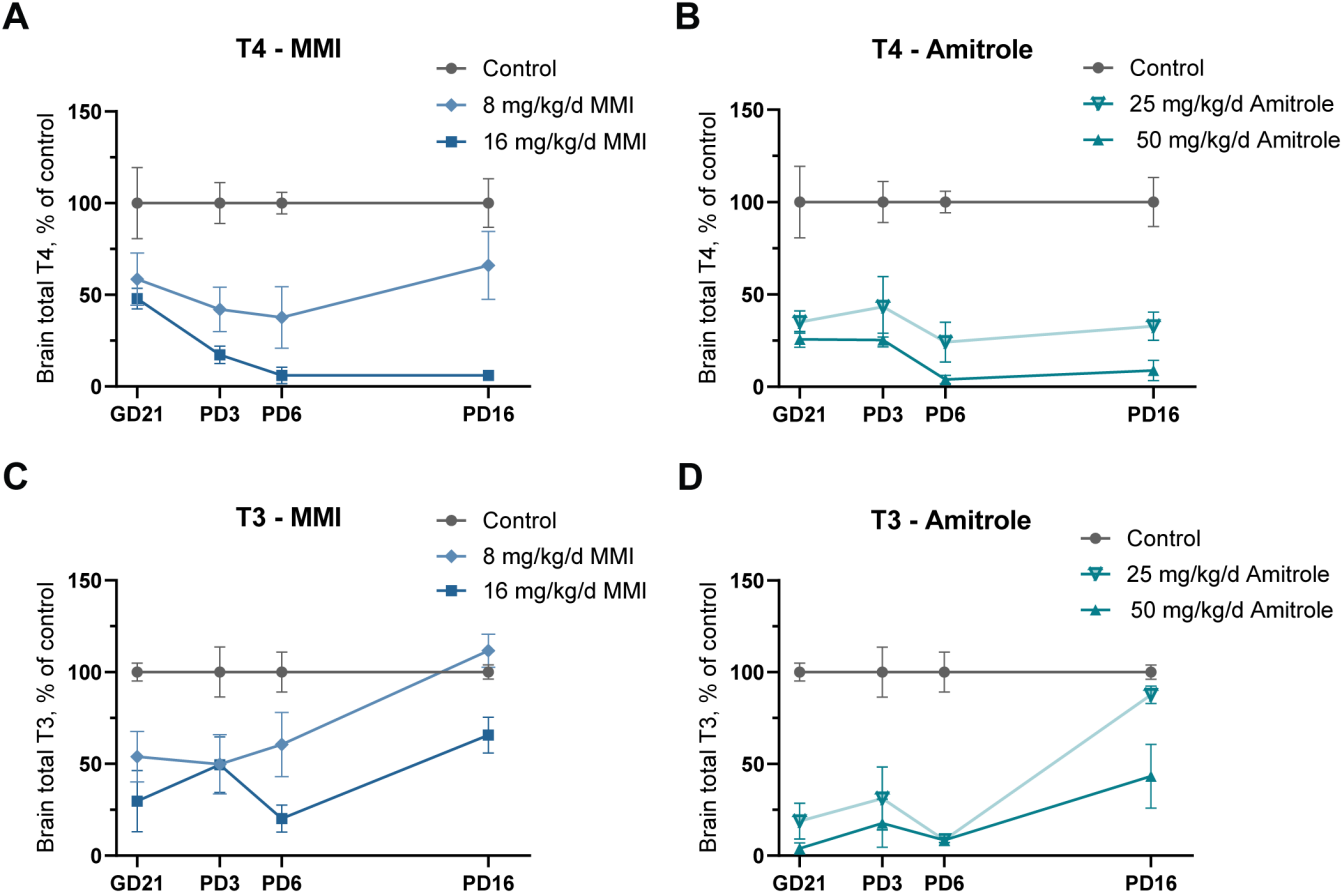
Gestational and lactational exposure to the thyroperoxidase inhibitors MMI and amitrole reduced offspring brain total T3 and T4 during development. Brain T4 and T3 concentrations as percentage of controls were reduced in both MMI **(A, C)** and amitrole **(B, D)** exposed offspring during the critical window for TH deficiency for heterotopia formation. TH concentrations during development followed different trajectories according to exposure group. Data are shown as group means ± SEM. Sample sizes and indications of statistical significance are given in **Supplementary Table S2**. GD, gestation day; LC-MS/MS, liquid chromatography/tandem mass spectrometry; mg/kg/d, mg/kg body weight/day; MMI, methimazole; PD, pup day; T3, tri-iodothyronine; T4, thyroxine; TH, thyroid hormone.

During the late fetal and early neonatal period, brain T4 and T3 concentrations were continuously suppressed to less than 50% of controls in high-dose MMI and both doses of amitrole (**Figure 3**; **Supplementary Table S2**). Suppressed brain T4 concentration was maintained in the high-dose groups on PD16 but recovery was evident by PD16 in the low-dose groups. Brain T3 concentrations were variable and the smaller effect sizes led to fewer statistically significant effects, most notable for the low-dose MMI where there was a lack of statistical significance at any age. Some recovery of brain T3 concentrations was evident by PD16, with statistically significant reductions seen only in animals exposed to the high-dose amitrole.

### 3.5 Mapping relationship of serum to brain TH concentrations

**Figure 4:**
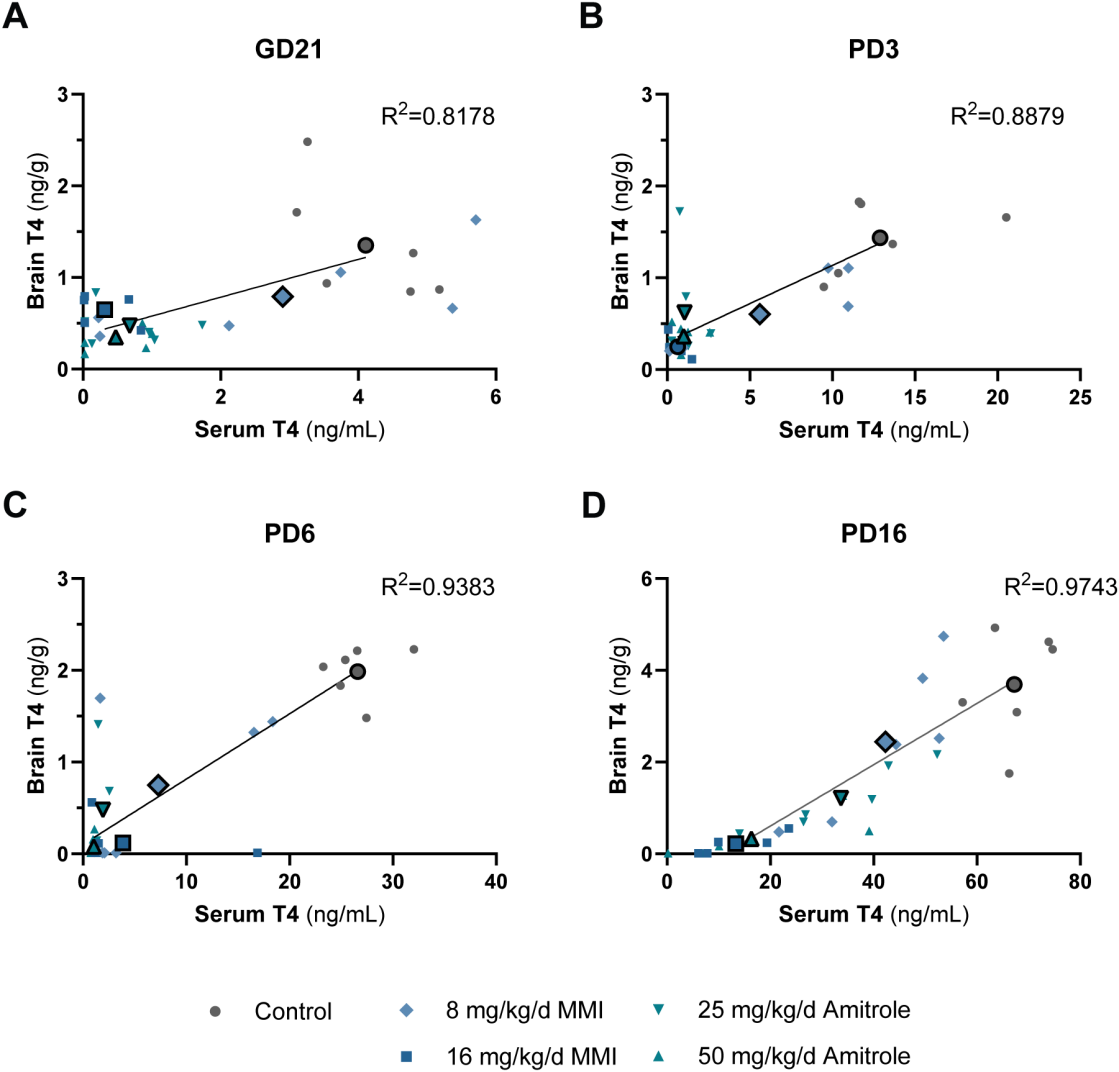
Relationship between brain and serum T4 concentrations in male offspring exposed to MMI or amitrole during development. Brain T4 concentrations were plotted against serum T4 concentrations for GD21 **(A)**, PD3 **(B)**, PD6 **(C)**, and PD16 **(D)**. The dataset represents the subset of individuals/litters for which both brain and serum TH concentrations were available. Brain T4 concentration was measured in one fetus or pup per litter and serum T4 concentrations are from the corresponding litter; in the fetuses, all male serum was pooled per litter; for PD3, PD6, and PD16, serum T4 was measured in a pool of one to two male pups. Small symbols indicate data per individual/litter and large symbols show means for each group for the subset of data depicted. A linear regression line was fitted on the group means of all five groups. *n* = 5–6. The full datasets, along with statistical significance, are shown in **Supplementary Table S2**. GD, gestation day; MMI, methimazole; PD, pup day; T4, thyroxine.

To examine the relationship between peripheral markers of TH status in serum and TH in the central nervous system, correlational analyses were performed. Brain and serum T4 concentrations were available for a subset of six litters/dose group. For these litters, concentrations of brain T4 (1 pup per litter) and serum T4 (pool of all male fetuses or of one to two male pups) were plotted for each age (**Figure 4**). These graphs showed the dramatic changes in serum T4 concentrations (note the *x*-axis range by age) during development and the changing relationship between brain and serum hormone concentrations. Simple linear regressions were significant for the correlations across the means for all groups (large symbols), while data on individual litters appeared variable (small symbols).

### 3.6 Periventricular heterotopia in postnatal offspring

**Figure 5:**
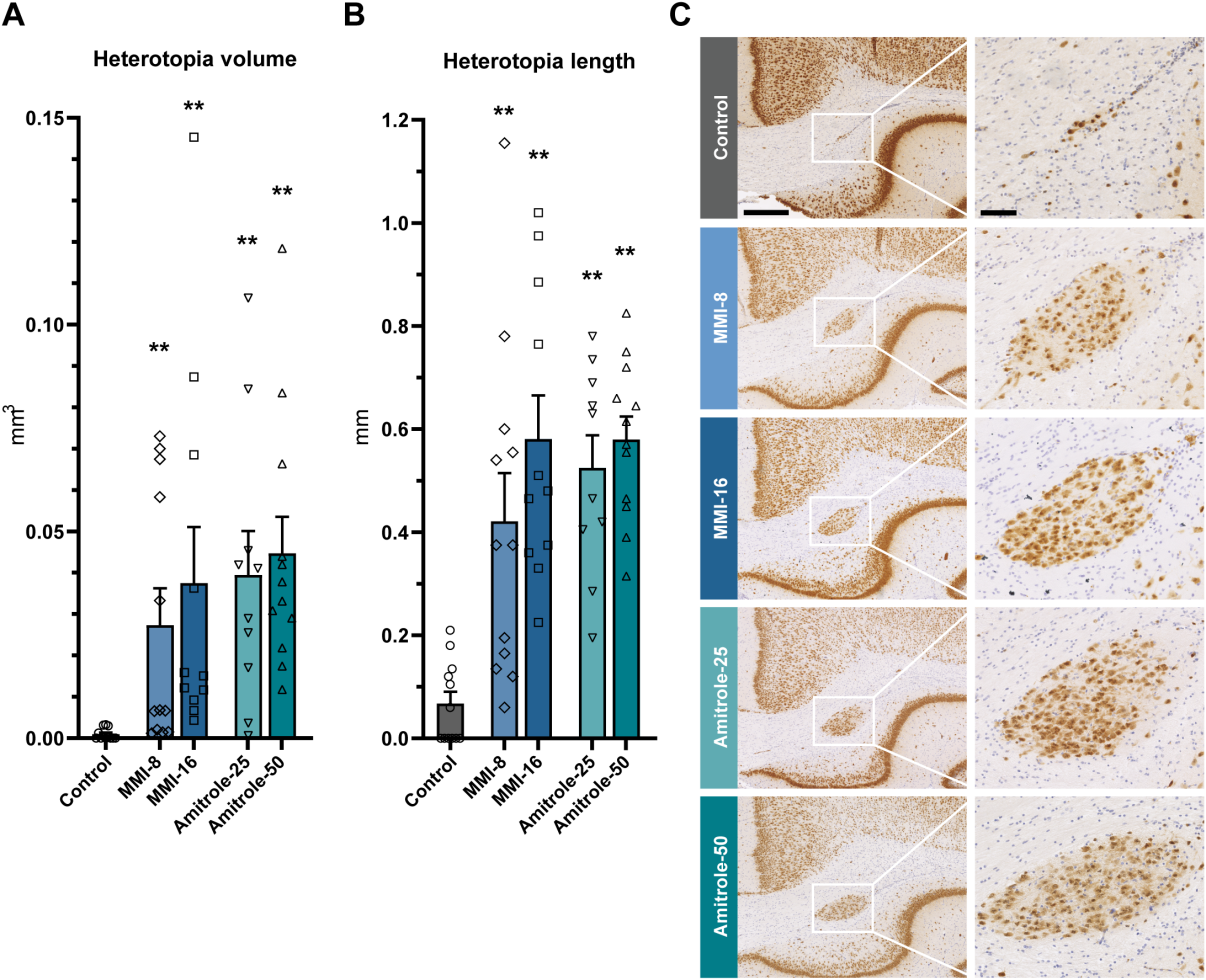
Periventricular heterotopia in 16-day-old male rat offspring exposed to thyroperoxidase inhibitors MMI and amitrole during development. Heterotopia volume **(A)** as well as the rostral–caudal length of heterotopia per hemisphere **(B)** were increased in all exposure groups. Data are shown as mean ± SEM, and individual datapoints are shown as symbols. *n* = 10–12. Representative sections with heterotopia **(C)** show overview images (left column, scale bars = 500 µm) and magnified areas (right column, scale bar = 100 µm). NeuN+ = brown (DAB), cell nuclei = blue (hematoxylin). Small neuronal clusters were found in 6 out of 12 control animals; the mean volume was 0.000958 mm^3^. Doses given in mg/kg/d. Ami, amitrole; MMI, methimazole; mg/kg/d, mg/kg body weight/day. **p < 0.01.

TH deficiency induced by MMI and amitrole was accompanied by heterotopia present in the brains of offspring assessed on PD16 (**Figure 5**). Statistically significant differences in both volume and length estimates of heterotopia were observed for all dose groups relative to controls. Considerable variability was evident in both metrics, but volume, a more precise estimate of overall size, provided a larger dynamic range and a clearer difference between controls and exposed animals. The heterotopia volume in animals exposed to MMI showed a bimodal distribution, as also observed in some hormone metrics, where some animals have large heterotopia and others have smaller, sometimes within the range of control animals.

**Figure 6:**
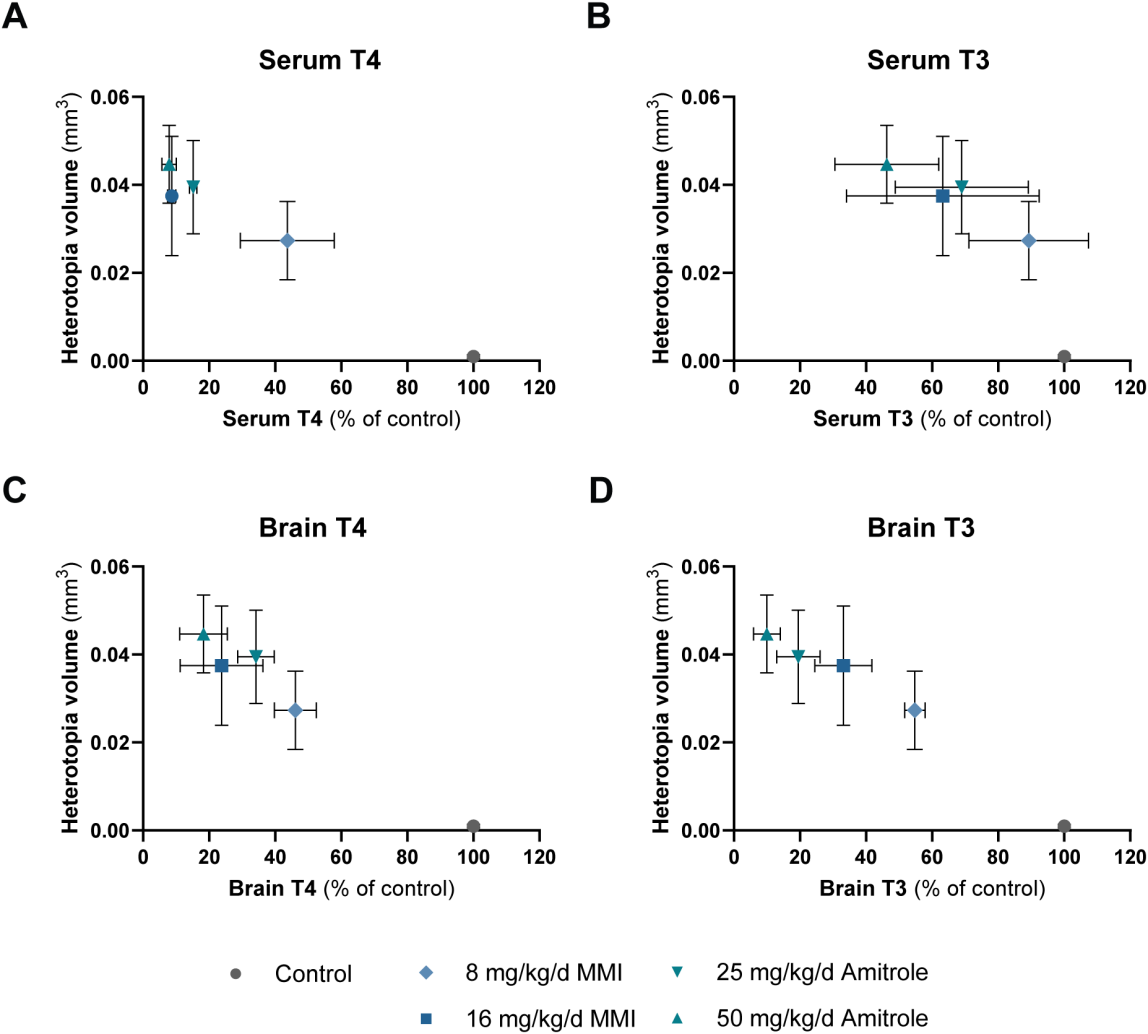
Relationship between heterotopia volume and TH levels in serum and brain from GD21 to PD6 in male offspring exposed to MMI and amitrole during development. Heterotopia forms as a consequence of reduced TH action throughout an entire critical window for TH deficiency from approximately GD19 to PD6. To get an estimate of the correlation between heterotopia and TH concentrations during the sensitive window, we correlated heterotopia volume to TH concentrations given as a mean of percent of control values for GD21, PD3, and PD6 for serum T4 **(A)**, serum T3 **(B)**, brain T4 **(C)**, and brain T3 **(D)**. Thus, the *x*-axes represent an estimation of the TH deficiency during the critical window. Importantly, this approach is reasonable with the present dataset, since TH concentration reductions are relatively constant throughout the period. If reductions had been restricted to only parts of the critical window, heterotopia formation cannot be expected. Mean heterotopia volume in the control group was 0.000958 mm^3^. *n* for TH concentrations specified in **Supplementary Table S2**; *n* = 10–12 for heterotopia. Data are shown as group means ± SEM for *y*- and *x*-axes (error bars for heterotopia in the control group fall within the symbol for the group). GD, gestation day; MMI, methimazole; PD, pup day; T3, tri-iodothyronine; T4, thyroxine; TH, thyroid hormone.

Heterotopia is believed to arise from brain TH deficiency between GD21 and PD6 (19, 22, 23). We therefore examined the relationship between TH status and mean heterotopia volume as a function of the average percent of control serum (**Figures 6A, B**) and brain TH concentrations (**Figures 6C, D**). Note that this approach is reasonable for these two chemicals since they induce similar degrees of hormone reductions during this timeframe. If reductions had been restricted to only parts of the critical window, heterotopia formation cannot be expected. The plots suggest similar relationships between TH concentrations and heterotopia for MMI and amitrole for these dose groups. Furthermore, the data are skewed to the left, with this study not informing on the relationship between heterotopia and smaller decrements in TH concentrations.

## 4 Discussion

In this study, we report altered serum TH concentrations in fetal and newborn rat pups following maternal exposure to two TH synthesis-inhibiting chemicals, one pharmaceutical, MMI, and one pesticide, amitrole. While MMI was designed to inhibit TPO, the triazole amitrole is a herbicide with unintentional TPO-inhibiting properties. At the dose levels used, amitrole was as effective as MMI, if not more effective, in reducing fetal thyroid gland function and lowering circulating and brain TH concentrations. This resulted in a clear adverse neurodevelopmental outcome for both compounds at both dose levels. Our findings replicate preliminary findings from our lab with amitrole and, to our knowledge, are the first full characterization of dose-dependent early-life reductions in serum and brain TH and heterotopia formation for both MMI and amitrole. These findings are also consistent with previous reports of heterotopia induced by PTU, MMI, and, recently, the environmental contaminant perchlorate and the radiocontrast agent iopanoic acid (7, 10, 19–26, 38). Collectively, the data serve to affirm that this brain defect in rat is a direct consequence of disrupted TH signaling.

### 4.1 Serum TH profiles differ in the dam, fetus, neonate, and postnatal pup

It is becoming increasingly clear that TH system-disrupting compounds can induce diverse serum TH effect patterns, which may vary with exposure conditions and life stage (6, 23, 39–41). There are many reasons for this, including mode of action, exposure route (gavage, transplacental, lactation, feed, and drinking water), ADME (absorption, distribution, metabolism, and excretion), toxicokinetics, and developmental stage at exposure. The data presented herein exemplify some of these points, particularly the low dose MMI group is instructive. Serum T4 concentrations in the fetuses and pups were relatively constantly suppressed in the high-dose groups. However, in the low-dose MMI, they varied by age with non-significant decreases in the fetuses, marked suppression in the early postnatal period, and recovery to approximately 60% of control levels in the PD16 pups. In these groups, some litters were affected, while others were not. This pattern was paralleled in the dams and coincided with affected fetuses having 5- to 10-fold higher serum MMI concentrations compared to non-responders in the same group [data reported in (32)]. High variability also persisted on PD3 and PD6, a factor that could contribute to the variance observed in heterotopia volumes assessed in pups on PD16, as discussed below. Collectively, these observations are important as they indicate that considerable between-litter variability may obscure these vulnerabilities; that TH profiles and sensitivities change dramatically with age; and that dose selection is critical in revealing these patterns.

### 4.2 Heterotopia assessment

The brains of pups were examined on PD16 for the presence of heterotopia. Both MMI and amitrole pups exhibited heterotopia with dose-dependent increases in size. This is consistent with earlier findings for PTU and perchlorate where both incidence and volume metrics indicated that the more severe the thyroid disruption, the more prevalent and larger the heterotopia (20, 26, 38). In our study, average heterotopia size in exposed animals was significantly greater than background levels observed in controls. However, we also observed substantial variability in both MMI- and amitrole-exposed groups—some animals falling within or near the control range, while others exhibiting large heterotopia. Some of this variability may be related to the observed variation in serum and brain TH concentration reductions. Addressing this possibility would require extensive studies with very large group sizes. Such studies should use frequent monitoring of serum and brain hormone concentrations and how it relates to heterotopia observed later in development, which is beyond the scope of our study. Despite some limitations of the current study, we detected heterotopia in pups from all exposure groups. This included the low-dose MMI group exhibiting the highest degree of variability in TH measures in serum and brain. We quantified heterotopia by both volume and rostral–caudal length. Our data suggest that volume-based quantification can offer a higher signal-to-noise ratio and greater dynamic range between controls and exposed animals. Volume estimation of NeuN‑stained sections is recommended for future studies, either by using stereological methods with a sufficient number of thin sections spanning the anterior–posterior extent of the heterotopia, or by assessing a consecutive series of thick, vibratome‑prepared sections, as described in (20, 21, 26).

### 4.3 TH deficiency and heterotopia formation

Heterotopia formation following exposure to TPO inhibitors has previously been documented for PTU and MMI. Numerous studies have examined PTU-induced heterotopia (7, 10, 19, 20, 22, 24–26) and characterized the associated serum and brain hypothyroidism (7, 10, 20, 22, 26, 42). In contrast, data on MMI-induced heterotopia are more limited (19, 25, 27). This is, to our knowledge, the first dose–response analysis of TH levels in serum and brain during the critical window of TH-dependent heterotopia formation. Together with findings on amitrole and recent findings of heterotopia formation after exposure to perchlorate (35, 38, 43) and iopanoic acid (23), these datasets provide compelling evidence on heterotopia formation. Collectively, they point to insufficient TH signaling in the developing fetal and neonatal brain. The compounds shown to induce heterotopia do so through different mechanisms: TPO inhibition (PTU, MMI, and amitrole), inhibition of the sodium/iodide symporter (NIS) (perchlorate), and inhibition of the deiodinase enzymes (iopanoic acid). In different ways, these compounds cause heterotopia by reducing TH signaling in the developing brain: by reducing TH synthesis in the thyroid gland or by reducing local deiodination of T4 to the active T3. As such, heterotopia formation is a direct consequence of impaired TH signaling in the fetal/newborn brain rather than compound-specific toxicity, reinforcing observations by Goodman and Gilbert (19) whereby concurrent T4 administration to PTU-exposed dams prevented the formation of a heterotopia in offspring. These findings underscore the inherent hazards associated with chemicals that disrupt the TH system and the need for improved testing and regulation of TH system-disrupting chemicals.

### 4.4 Advancing AOP development for thyroid hormone system disruption and neurodevelopmental outcomes

In the European Union, a chemical can be regulated as an endocrine disruptor if an endocrine mode of action can be documented, an adverse effect is observed, and a biologically plausible link between the two can be established (44). These key elements of identification of endocrine disruptors are facilitated by engaging the adverse outcome pathway (AOP) framework. An AOP describes a causal pathway between a mechanism, a so-called molecular initiating event (MIE), and adverse outcomes (AOs) through a series of measurable key events (KEs) and key event relationships (KERs). For the TH system, there is a growing network of AOPs that are OECD endorsed or under development. One of them is the externally reviewed and endorsed AOP-42 “Inhibition of thyroperoxidase and subsequent adverse neurodevelopmental outcomes in mammals”. This AOP describes how TPO inhibition leads to altered hippocampal anatomy and decreased cognitive function (45). The data presented herein support and strengthen the knowledge base on the intermediate KE “thyroxine (T4) in serum, decreased” (KE-281) and “thyroxine (T4) in neuronal tissue, decreased” (KE-280) and confirm heterotopia formation as a downstream AO of reduced TH concentrations in serum and brain. Additionally, these data inform ongoing efforts to expand the suite of adverse outcomes following developmental TH system disruption in thyroid gland, brain, and reproductive organs (28–33).

## 5 Conclusions

Periventricular heterotopia is an apical endpoint demonstrating developmental neurotoxicity following exposure to TH system-disrupting compounds. It is a robust measure that clearly illustrates how chemicals can induce severe and irreversible adverse effects on brain development by affecting fetal and early postnatal TH signaling. As an environmental chemical, amitrole highlights the inherent hazards associated with exposure to compounds with unintended TPO-inhibiting properties. Therefore, it is critical that chemical testing and risk assessment account for the most sensitive populations and developmental windows. This includes thorough evaluation of TH system disruption in dams and early life stages and assessments of TH in serum, brain, and other tissues throughout development to reflect the dynamic nature of the TH system and brain development.

## Data Availability

The raw data supporting the conclusions of this article will be made available by the authors, without undue reservation.
