# Supplementary material for: Maternal exposure to the thyroperoxidase-inhibiting pesticide amitrole induces hypothyroidism and developmental neurotoxicity in the rat brain
Source: Front Endocrinol (Lausanne). 2026 May 12;17:1823237. doi: 10.3389/fendo.2026.1823237 (PMC13201139; doi:10.3389/fendo.2026.1823237)
Supplement: Supplementary file 1 [file SupplementaryFile1.docx]

**Supplementary materials**

*Supplementary materials to Ramhøj et al.,* *“Maternal exposure to the thyroperoxidase-inhibiting pesticide amitrole induces hypothyroidism and developmental neurotoxicity in the rat brain”*

**Table S1:** Dam and litter toxicity data from gestational day (GD) 21.

| ***Dams and litters*** | **Control** | **MMI 8 mg/kg** | **MMI 16 mg/kg** | **Amitrole 25 mg/kg** | **Amitrole 50 mg/kg** |
| --- | --- | --- | --- | --- | --- |
| No. of pregnant dams | 6 | 6 | 6 | 6 | 6 |
| ***Dam body weight*** |  |  |  |  |  |
| Maternal weight GD21 | 396 ± 25 | 374 ± 26 | **350 ± 24*** | 380 ± 33 | 381 ± 18 |
| Maternal GD21 bw without uterus (g) | 302 ± 15 | 281 ± 16 | **261 ± 19**** | 288 ± 25 | 297 ± 12 |
| Maternal bw gain GD7-GD21 (g) | 136 ± 21 | 118 ± 16 | **102 ±12**** | 125 ± 18 | 113 ± 13 |
| Maternal liver weight (g)^1^ | 14.33 ± 1.27 | 12.67 ± 1.08 | **10.86 ± 0.79*** | 13.78 ± 1.90 | 12.90 ± 1.03 |
| ***Litters*** |  |  |  |  |  |
| Litter size, fetuses GD21 (no.) | 14.7 ± 1.9 | 14.8 ± 2.6 | 15.7 ± 3.3 | 14.7 ± 2.3 | 14.5 ± 1.4 |
| Post-implantation loss (prenatal mortality) (%) | 5.9 ± 5.2 | 4.7 ± 5.9 | 2.4 ± 3.7 | 3.2 ± 3.6 | 4.7 ± 5.8 |
| Resorptions (no.) | 0.8 ± 0.8 | 0.7 ± 0.8 | 0.3 ± 0.5 | 0.5 ± 0.6 | 0.7 ± 0.8 |
| % Males / Females | 55 / 45 ± 10 | 52 / 48 ± 12 | 52 / 48 ± 11 | 56 / 44 ± 8 | 45 / 55 ± 19 |
| ***Offspring*** |  |  |  |  |  |
| Male fetal weight (g)^2^ | 4.3 ± 0.4 | 4.2 ± 0.2 | 3.8 ± 0.3 | 4.0 ± 0.4 | **3.8 ± 0.2*** |
| Female fetal weight (g)^2^ | 4.0 ± 0.3 | 4.0 ± 0.2 | 3.5 ± 0.3 | 3.8 ± 0.4 | 3.6 ± 0.2 |

^1^Body weight was included as a covariate in the statistical analysis

^2^Based on litter means. Number of fetuses included as a covariate in the statistical analysis

Data represent group means. based on dams or litter means ± SD. *p < 0.05. **p < 0.01. Statistically significant values shown in bold.

Doses given in mg/kg body weight(bw)/day(d)

**Table S2:** TH system disruption in dams, fetuses and postnatal offspring.

|  | **Control** | **MMI 8 mg/kg** | **MMI 16 mg/kg** | **Amitrole 25 mg/kg** | **Amitrole 50 mg/kg** |
| --- | --- | --- | --- | --- | --- |
| ***Dam (no.)*** | 6 | 6 | 6 | 6 | 6 |
| ***Dam GD15*** |  |  |  |  |  |
| T4^1^, ng/mL | 22.9 ± 4.8 | 19.4 ± 3.4 | **6.4 ± 2.1**** | **15.2 ± 1.9**** | **7.2 ± 3.4**** |
| T3^1^, ng/mL | 0.57 ± 0.07 | 0.58 ± 0.07 | 0.52 ± 0.07 | 0.50 ± 0.06 | **0.37 ± 0.08**** |
| *TSH, pg/mL* | 489 ± 258 | 1810 ± 2360 | **2566 ± 1645**** | **1896 ± 1513*** | **2485 ± 998**** |
| ***Dams GD21*** |  |  |  |  |  |
| Dam thyroid weight (mg)^1^ | 12.9 ± 2.7 | 12.4 ± 2.7 | 17.8 ± 4.8 | 15.2 ± 6.1 | **24.9 ± 9.6*** |
| Dam thyroid weight as ‰ of body weight | 0.033 ± 0.007 | 0.034 ± 0.008 | 0.051 ± 0.015 | 0.041 ± 0.018 | **0.0646 ± 0.022**** |
| Dam T4, ng/mL | 14.2 ± 2.1 | 9.1 ± 4.5 | **0.4 ± 0.2**** | 6.6 ± 3.2 | **0.7 ± 0.7**** |
| Dam T3, ng/mL | 0.58 ± 0.07 | 0.48 ± 0.10 | **0.29 ± 0.06**** | 0.48 ± 0.10 | **0.27 ± 0.04**** |
| **Fetal serum hormones**^3^ |  |  |  |  |  |
| ***Male fetus GD21 (no.)*** | 6 | 6 | 6 | 6 | 6 |
| T4, ng/mL | 4.1 ± 0.9 | 2.9 ± 2.4 | **0.4 ± 0.4**** | **0.7 ± 0.7*** | **0.5 ± 0.5**** |
| ***Female fetus GD21 (no.)*** | 6 | 6 | 6 | 6 | 6 |
| T4, ng/mL | 4.5 ± 1.1 | 3.1 ± 2.4 | **0.5 ± 0.5**** | **0.6 ± 0.5*** | **0.4 ± 0.5**** |
| **Postnatal offspring serum hormones (no. litters)**^4^ | 12 | 12 | 11 | 11 | 12 |
| ***Male pup PD3 (no.)*** | 12 | 11 | 9 | 10 | 8 |
| T4, ng/mL | 12.7 ± 3.1 | **4.8 ± 5.7**** | **0.8 ± 0.5 **** | **1.6 ± 1.2 **** | **1.0 ± 0.7**** |
| T3, ng/mL | 0.26 ± 0.07 | 0.22 ± 0.12 | **0.07 ± 0.03 **** | **0.11 ± 0.04 **** | **0.09 ± 0.05**** |
| ***Female pup PD3 (no.)*** | 9 | 10 | 9 | 11 | 9 |
| T4, ng/mL | 13.9 ± 2.8 | **4.2 ± 5.7 **** | **0.8 ± 0.5**** | **2.5 ± 2.6**** | **1.1 ± 0.6**** |
| T3, ng/mL | 0.27 ± 0.06 | **0.14 ± 0.07*** | **0.06 ± 0.03**** | **0.15 ± 0.08 *** | **0.09 ± 0.05**** |
| ***Male pup PD6 (no.)*** | 12 | 12 | 11 | 10 | 11 |
| T4, ng/mL | 25.7 ± 3.4 | **5.7 ± 6.5**** | **2.6 ± 4.7**** | **4.4 ± 4.0**** | **1.0 ± 0.2**** |
| T3, ng/mL | 0.40 ± 0.08 | **0.24 ± 0.13*** | **0.17 ± 0.19**** | 0.26 ± 0.20 | **0.10 ± 0.09**** |
| TSH, pg/ml (no.) | 932 ± 244 (9) | **3429 ± 1578 (8)**** | **3667 ± 1004 (7)**** | **4254 ± 1966 (6)**** | **4091 ± 1139 (8)**** |
| ***Female pup PD6 (no.)*** | 11 | 12 | 11 | 11 | 10 |
| T4, ng/mL | 22.4 ± 3.8 | **7.0 ± 7.7**** | **1.2 ± 0.8**** | **7.0 ± 5.6**** | **0.9 ± 0.4**** |
| T3, ng/mL | 0.33 ± 0.05 | 0.28 ± 0.11 | **0.12 ± 0.05**** | 0.30 ± 0.19 | **0.08 ± 0.04**** |
| TSH, pg/ml (no.) | 1036 ± 560 (10) | **4511 ± 2069 (11)**** | **3717 ± 1679 (10)*** | **5066 ± 3024 (10)**** | **4910 ± 2968 (7)**** |
| ***Male pup PD16 (no.)*** | 12 | 12 | 11 | 11 | 12 |
| T4, ng/mL | 65.7 ± 6.8 | **39.5 ± 11.9 **** | **9.8 ± 6.4**** | **37.9 ± 12.8**** | **10.3 ± 13.0**** |
| T3, ng/mL | 1.00 ± 0.11 | 0.85 ± 0.10 | **0.63 ± 0.20**** | 0.83 ± 0.13 | **0.52 ± 0.25**** |
| **Offspring brain hormones** |  |  |  |  |  |
| ***Male fetus GD21 (no.)*** | 6 | 6 | 5 | 6 | 6 |
| T4, ng/g | 1.35 ± 0.64 | 0.79 ± 0.48 | 0.65 ± 0.17 | **0.47 ± 0.20**** | **0.35 ± 0.14**** |
| T3, ng/g. ND set to LOD = 0.01 ng/g | 1.14 ± 0.14 | 0.61 ± 0.39 | **0.34 ± 0.46 (3/6 ND)*** | **0.21 ± 0.25 (2/5 ND)*** | **0.05 ± 0.08 (5/6 ND)**** |
| ***Male pup PD3 (no.)*** | 6 | 6 | 4 | 6 | 6 |
| T4, ng/g | 1.44 ± 0.40 | **0.60 ± 0.43*** | **0.25 ± 0.14**** | **0.62 ± 0.58*** | **0.36 ± 0.13**** |
| T3, ng/g. ND set to LOD = 0.01 ng/g | 2.59 ± 0.86 | 1.27 ± 1.01 | 1.27 ± 0.95 | **0.80 ± 1.07 (1/6 ND)*** | **0.45 ± 0.83 (2/6 ND)**** |

| ***Male pup PD6 (no.)*** | 6 | 6 | 6 | 6 | 6 |
| --- | --- | --- | --- | --- | --- |
| T4, ng/g. ND set to LOD = 0.01 ng/g | 1.99 ± 0.29 | **0.75 ± 0.82 (3/6 ND)**** | **0.12 ± 0.22 (4/6 ND)**** | **0.48 ± 0.52 (1/6 ND)*** | **0.08 ± 0.11 (4/6 ND)**** |
| T3, ng/g | 3.39 ± 0.90 | 2.05 ± 1.45 | **0.69 ± 0.62**** | **0.29 ± 0.12**** | **0.28 ± 0.25**** |
| ***Male pup PD16 (no.)*** | 6 | 6 | 6 | 6 | 6 |
| T4, ng/g. ND set to LOD = 0.01 ng/g | 3.69 ± 1.20 | 2.44 ± 1.68 | **0.22 ± 0.20 (2/6 ND)**** | 1.21 ± 0.70 | **0.33 ± 0.50 (3/6 ND)**** |
| T3, ng/g. ND set to LOD = 0.01 ng/g | 6.11 ± 0.59 | 6.83 ± 1.36 | 4.01 ± 1.46 | 5.36 ± 0.73 | **2.65 ± 2.61 (1/6 ND)**** |

^1^GD15 T4 and T3 serum concentration was measured by radioimmunoassay (RIA). All other TH measurements were measured by LC-MS/MS.  ^2^ Body weight was included as a covariate in the statistical analysis.

^3^Fetal serum chemical concentrations and steroid hormones have been published previously (Draskau et al., 2024).

^4^Other data from these litters have been published in (Ramhøj et al., 2022, 2024 and Draskau et al., 2024).
Data represents group means ± SD. * p < 0.05, ** p < 0.01. Statistically significant values shown in bold.
ND: non-detect.

Doses given in mg/kg body weight (bw)/day (d).
T3: 1 ng/mL = 1.538 nmol/L, T4: 1 ng/mL = 1.287 nmol/L.
